# Inaugural message from the new Co-Editor-in-Chief

**DOI:** 10.1038/s41377-026-02221-9

**Published:** 2026-02-10

**Authors:** Martin J. Booth

**Affiliations:** Department of Engineering Science, University of Oxford, Parks Road, Oxford, OX1 3PJ UK

**Keywords:** Other photonics, Optics and photonics

It is a great honour to assume the role of Co-Editor-in-Chief of *Light: Science & Applications* (hereinafter referred to as *Light*). I would like to begin by expressing my sincere gratitude to Professor Jianlin Cao and the founding editors for their vision and insight in establishing this journal. Their efforts laid the foundations for what has become one of the most influential platforms in our field. I am also deeply thankful to my predecessor, Professor Xi-Cheng Zhang, for his years of leadership and dedication, as I am to continuing Co-Editor-in-Chief Yun-Feng Xiao for his ongoing stewardship of the journal. One must also recognise the immense contributions of Professor Yuhong Bai and the entire editorial team for their continued commitment to the journal’s mission.

Today, *Light* plays a prominent role in disseminating the highest quality research to the global scientific community. The journal reflects the remarkable breadth and vitality of our field, covering topics that range from microscopes to telescopes, from laser manufacturing to quantum sensing, from green photonics to biomedical optics, amongst many others. This diversity of the science of light and its varied applications is one of the journal’s greatest strengths and a true reflection of the central role that light plays in modern science and technology.

The journal’s aim is to publish original articles and reviews that are of high quality, high interest and far-reaching consequence from all aspects of optics and photonics, including basic, applied and engineering research and applications. It has always been important to me that editorial decisions for this journal are based solely upon the significance of the research to the field, as assessed by expert reviewers and editors. It is this ethos that builds respect in the field and will be the key to *Light*’s future success. I know that such success is only possible through those experts volunteering their valuable time to uphold the highest standards as a service to the scientific community. For this commitment, we all owe our thanks.

I very much look forward to supporting and working with the journal’s vibrant community of researchers, authors, editors, reviewers, and readers as we continue to advance the science of light and develop its applications together.

